# Supplementary material for: Combining supervised and unsupervised analyses to quantify behavioral phenotypes and validate therapeutic efficacy in a triple transgenic mouse model of Alzheimer’s disease
Source: Biomed Pharmacother. Author manuscript; Available in PMC 2025 Jan 23. (PMC11755788; doi:10.1016/j.biopha.2024.117718)
Supplement: 1 [file NIHMS2042844-supplement-1.docx]

**Supplementary Materials**

**Supplementary Figure 1.** Analysis of syllables generated with Keypoint MoSeq. Frequency of syllables in A) young, B) old, and C) aged 3xTg-AD mice compared to WT mice. Syllable transition graphs of D) young, E) old, and C) aged 3xTg-AD mice compared to WT mice. Syllable frequencies of old mice during the first 10 minutes of the moth stimulus (G), the first set of moving lines in the home quadrant (H), and the second set of moving lines in the home quadrant (I).

**Supplementary Figure 2.** Mouse, cage, and stimuli markers used in the DeepLabCut model. 27 out of 48 markers were utilized for analysis in the current study.

**Supplementary File 1.** PowerPoint Presentation of 22-hour behavioral assay.

**Supplementary File 2.** Fiji/ImageJ macro for the tracking and analysis of mouse behavior in an 8-cage imaging system.

**Supplementary File 3.** Raw data with measures of behavior in Fiji, DeepLabCut, and MoSeq. Columns d0-d63 = duration of motion sequences (some motion sequences, such as d17-19, are automatically removed by the software). Columns f0-f63 = frequency of motion sequences. Columns M1-Win Partner = behaviors measured in DeepLabCut. Columns M1-Out = behaviors measured in Fiji. Each row represents an individual mouse.

**Supplementary Table 1.** CatWalk gait analysis.

**Supplementary Table 2.** Behavioral parameters measured using Fiji.

**Supplementary Table 3.** Behavioral parameters measured using DeepLabCut.

**Supplementary Table 4.** Top behavioral syllables identified using Keypoint MoSeq
