# Supplementary material for: Combining supervised and unsupervised analyses to quantify behavioral phenotypes and validate therapeutic efficacy in a triple transgenic mouse model of Alzheimer’s disease
Source: Biomed Pharmacother. Author manuscript; Available in PMC 2025 Jan 23. (PMC11755788; doi:10.1016/j.biopha.2024.117718)
Supplement: 2 [file NIHMS2042844-supplement-2.docx]

**Supplementary Table 1.** CatWalk gait analysis.

|  |  | Genotype | | Age | |
| --- | --- | --- | --- | --- | --- |
| Measure | Type | Young | Old | WT | AD |
| Print length | LF | *↓ | NS | NS | NS |
|  | RF | *↓ | NS | NS | NS |
|  | LH | *↓ | NS | NS | NS |
|  | RH | NS | **↓ | NS | NS |
| Toe spread | LH | NS | NS | NS | NS |
|  | RH | NS | NS | NS | NS |
| Intermediate toe spread | LF | NS | NS | NS | NS |
|  | RF | NS | NS | NS | NS |
|  | LH | NS | NS | ***↓ | ***↓ |
|  | RH | NS | NS | ***↓ | ***↓ |
| Print area | LF | *↑ | NS | *↑ | NS |
|  | RF | NS | NS | *↑ | NS |
|  | LH | NS | NS | NS | NS |
|  | RH | NS | NS | NS | NS |
| Maximum contact area | LF | **↑ | NS | *↑ | NS |
|  | RF | NS | NS | *↑ | NS |
|  | LH | NS | NS | NS | NS |
|  | RH | NS | NS | NS | NS |
| Mean intensity | LF | NS | ***↑ | NS | ***↑ |
|  | RF | NS | ***↑ | *↑ | ***↑ |
|  | LH | NS | NS | NS | **↑ |
|  | RH | NS | NS | NS | ***↑ |
| Minimum intensity | LF | NS | ***↑ | NS | ***↑ |
|  | RF | NS | ***↑ | NS | ***↑ |
|  | LH | NS | NS | NS | **↑ |
|  | RH | NS | NS | NS | **↑ |
| Stand | LF | NS | NS | NS | NS |
|  | RF | NS | NS | NS | NS |
|  | LH | NS | NS | NS | NS |
|  | RH | NS | NS | NS | *↑ |
| Paw angle body axis | LF | **↑ | NS | NS | NS |
|  | RF | *↑ | NS | NS | NS |
|  | LH | NS | *↓ | NS | *↓ |
|  | RH | NS | NS | NS | NS |
| Step cycle | LF | NS | NS | NS | NS |
|  | RF | NS | NS | NS | NS |
|  | LH | NS | NS | NS | NS |
|  | RH | NS | NS | NS | *↑ |
| Support | Lateral | NS | NS | NS | NS |
|  | Girdle | NS | NS | NS | NS |
|  | Diagonal | NS | NS | NS | NS |
|  | Four | NS | NS | NS | NS |
|  | Three | NS | NS | NS | NS |
|  | Single | NS | NS | NS | NS |
|  | Zero | NS | NS | NS | NS |
| Base of support -front paws | | NS | NS | NS | NS |
| Base of support -hind paws | | NS | NS | NS | *↑ |
| Duration |  | NS | NS | NS | NS |
| Cadence |  | NS | NS | NS | NS |
| Number of steps | | *↓ | NS | **↓ | NS |
| Step sequence | | *↓ | NS | ***↓ | NS |
| ↑ and ↓ denote an increase or decrease in 3xTg-AD mice compared to WT mice (Genotype), or an increase or decrease in old mice compared to young mice (Age), respectively. | | | | | |
