# Supplementary material for: Combining supervised and unsupervised analyses to quantify behavioral phenotypes and validate therapeutic efficacy in a triple transgenic mouse model of Alzheimer’s disease
Source: Biomed Pharmacother. Author manuscript; Available in PMC 2025 Jan 23. (PMC11755788; doi:10.1016/j.biopha.2024.117718)
Supplement: 3 [file NIHMS2042844-supplement-3.docx]

***Supplementary Table 2. Behavioral parameters measured using Fiji***

| Behavioral measure | Description |
| --- | --- |
| M1 | Movement during the first hour |
| MD | Movement during daytime |
| M7 | Movement in the 7^th^ hour (i.e. the first hour of nighttime) |
| MN | Movement during nighttime |
| N-D | The difference between nighttime and daytime movement (MN-MD) |
| AM | Acclimation to the cage, measured in % movement |
| AS | Acclimation to the cage, measured in % stretch-attend posture (SAP) |
| HMM | Habituation to moth stimulus, measured in % movement |
| HL1M | Habituation to 1^st^ set of moving lines, measured in % movement |
| HL2M | Habituation to 2^nd^ set of moving lines, measured in % movement |
| Home | % time the mouse is in the home quadrant |
| Wall | % time the mouse is in the wall quadrant |
| Food | % time the mouse is in the food quadrant |
| Window | % time the mouse is in the window quadrant |
| Out | Sum of Wall and Window. |
