# Supplementary material for: Combining supervised and unsupervised analyses to quantify behavioral phenotypes and validate therapeutic efficacy in a triple transgenic mouse model of Alzheimer’s disease
Source: Biomed Pharmacother. Author manuscript; Available in PMC 2025 Jan 23. (PMC11755788; doi:10.1016/j.biopha.2024.117718)
Supplement: 4 [file NIHMS2042844-supplement-4.docx]

***Supplementary Table 3. Behavioral parameters measured using DeepLabCut***

| Behavioral measure | Description |
| --- | --- |
| M1 | Movement during the first hour |
| MD | Movement during daytime |
| M7 | Movement in the 7^th^ hour (i.e. the first hour of nighttime) |
| MN | Movement during nighttime |
| MN-D | The difference between nighttime and daytime movement (MN-MD) |
| Sc1 | Scoot during the first hour |
| ScD | Scoot during daytime |
| Sc7 | Scoot in the 7^th^ hour (i.e. the first hour of nighttime) |
| ScN | Scoot during nighttime |
| ScN-D | The difference between nighttime and daytime scoot (ScN-ScD) |
| B1 | Burst during the first hour |
| BD | Burst during daytime |
| B7 | Burst in the 7^th^ hour (i.e. the first hour of nighttime) |
| BN | Burst during nighttime |
| BN-D | The difference between nighttime and daytime burst (BN-BD) |
| E1 | Escape during the first hour |
| ED | Escape during daytime |
| E7 | Escape in the 7^th^ hour (i.e. the first hour of nighttime) |
| EN | Escape during nighttime |
| EN-D | The difference between nighttime and daytime escape (EN-ED) |
| Sp1 | Speed during the first hour |
| SpD | Speed during daytime |
| Sp7 | Speed in the 7^th^ hour (i.e. the first hour of nighttime) |
| SpN | Speed during nighttime |
| SpN-D | The difference between nighttime and daytime speed (SpN-SpD) |
| AM | Acclimation to the cage, measured in % movement |
| ASc | Acclimation to the cage, measured in % scoot |
| AB | Acclimation to the cage, measured in % burst |
| AE | Acclimation to the cage, measured in % escape |
| ASp | Acclimation to the cage, measured in speed (pixels) |
| HMM | Habituation to moth stimulus, measured in % movement |
| HL1M | Habituation to 1^st^ set of moving lines, measured in % movement |
| HL2M | Habituation to 2^nd^ set of moving lines, measured in % movement |
| HMSc | Habituation to moth stimulus, measured in % scoot |
| HL1Sc | Habituation to 1^st^ set of moving lines, measured in % scoot |
| HL2Sc | Habituation to 2^nd^ set of moving lines, measured in % scoot |
| HMB | Habituation to moth stimulus, measured in % burst |
| HL1B | Habituation to 1^st^ set of moving lines, measured in % burst |
| HL2B | Habituation to 2^nd^ set of moving lines, measured in % burst |
| HME | Habituation to moth stimulus, measured in % escape |
| HL1E | Habituation to 1^st^ set of moving lines, measured in % escape |
| HL2E | Habituation to 2^nd^ set of moving lines, measured in % escape |
| HMSp | Habituation to moth stimulus, measured in speed (pixels) |
| HL1Sp | Habituation to 1^st^ set of moving lines, measured in speed (pixels) |
| HL2Sp | Habituation to 2^nd^ set of moving lines, measured in speed (pixels) |
| Home | % time the mouse is inside the red hut |
| Peek | % time the mouse is inside the red hut with its head outside the hut |
| Window | % time the mouse is within 40 pixels of the window |
| Wall | Closest distance from any cage wall (pixels) |
| Food | Closest distance from any food pellet (pixels) |
| DWindow | Distance from the window (pixels) |
| Window Partner | % time spent near the window when partner mouse is also near the window |
| Speed: pixels moved. Movement: speed > 1.5px. Scoot: 11px > speed > 1.5px. Burst: speed > 10px. Escape: speed > 30px. | |
