# Supplementary material for: Combining supervised and unsupervised analyses to quantify behavioral phenotypes and validate therapeutic efficacy in a triple transgenic mouse model of Alzheimer’s disease
Source: Biomed Pharmacother. Author manuscript; Available in PMC 2025 Jan 23. (PMC11755788; doi:10.1016/j.biopha.2024.117718)
Supplement: 5 [file NIHMS2042844-supplement-5.docx]

***Supplementary Table 4. Top behavioral syllables identified using Keypoint MoSeq***

| Syllable ID | Label | Description |
| --- | --- | --- |
| 0 | groom_hunch | Hunched grooming |
| 1 | burst_turn | Turn 180 and burst across cage length |
| 2 | hunch_look | Hunch, pause, and look in one direction |
| 3 | burst_short | Sudden, agitated, fast burst(s) in a linear direction |
| 4 | freeze_look | Freeze and look around |
| 5 | rear | Rear and look up, while staying in place |
| 6 | stretch_burst | Stretch and take one long step |
| 7 | groom_quick | Grooming in quick separate bursts while looking around |
| 8 | burst_home | Burst and go towards the hut |
| 9 | groom_small | Slow grooming |
| 10 | rear_climb | Rear and/or climb an object or wall |
| 11 | stretch_sit | Stretch upper body forward while sitting and staying in place |
| 12 | ball | Hunched, moving minimally |
| 13 | curl | Stop moving and curl |
| 14 | rear_fast | Rapid rearing around the cage |
| 15 | burst_single | Quick burst away after staying in the same spot |
| 16 | stay | Stay in the same location with minimal movement, not hunched |
| 20 | food | Interact with the food or food plate (i.e. Grab pellet) |
| 21 | stretch | Stretch in place while standing or right after walking |
| 24 | groom_tail | Grooming the tail |
| 27 | loop | Burst away and then circle back to the same spot |
| 28 | sleep | Curled and asleep |
| 31 | groom_still | Grooming while staying still |
| 34 | turn_behind | Turn and/or look behind with a quick posterior groom |
| 42 | turn | Turn while staying in place |
| 46 | look | Look in different directions |
| 50 | turn_away | Turn away and move in opposite direction |
| 61 | freeze_hunch | Stop moving and stay hunched |
| 63 | small_burst | Small movements |
