# Supplementary figures and images for: Combining supervised and unsupervised analyses to quantify behavioral phenotypes and validate therapeutic efficacy in a triple transgenic mouse model of Alzheimer’s disease
Source: Biomed Pharmacother. Author manuscript; Available in PMC 2025 Jan 23. (PMC11755788; doi:10.1016/j.biopha.2024.117718)

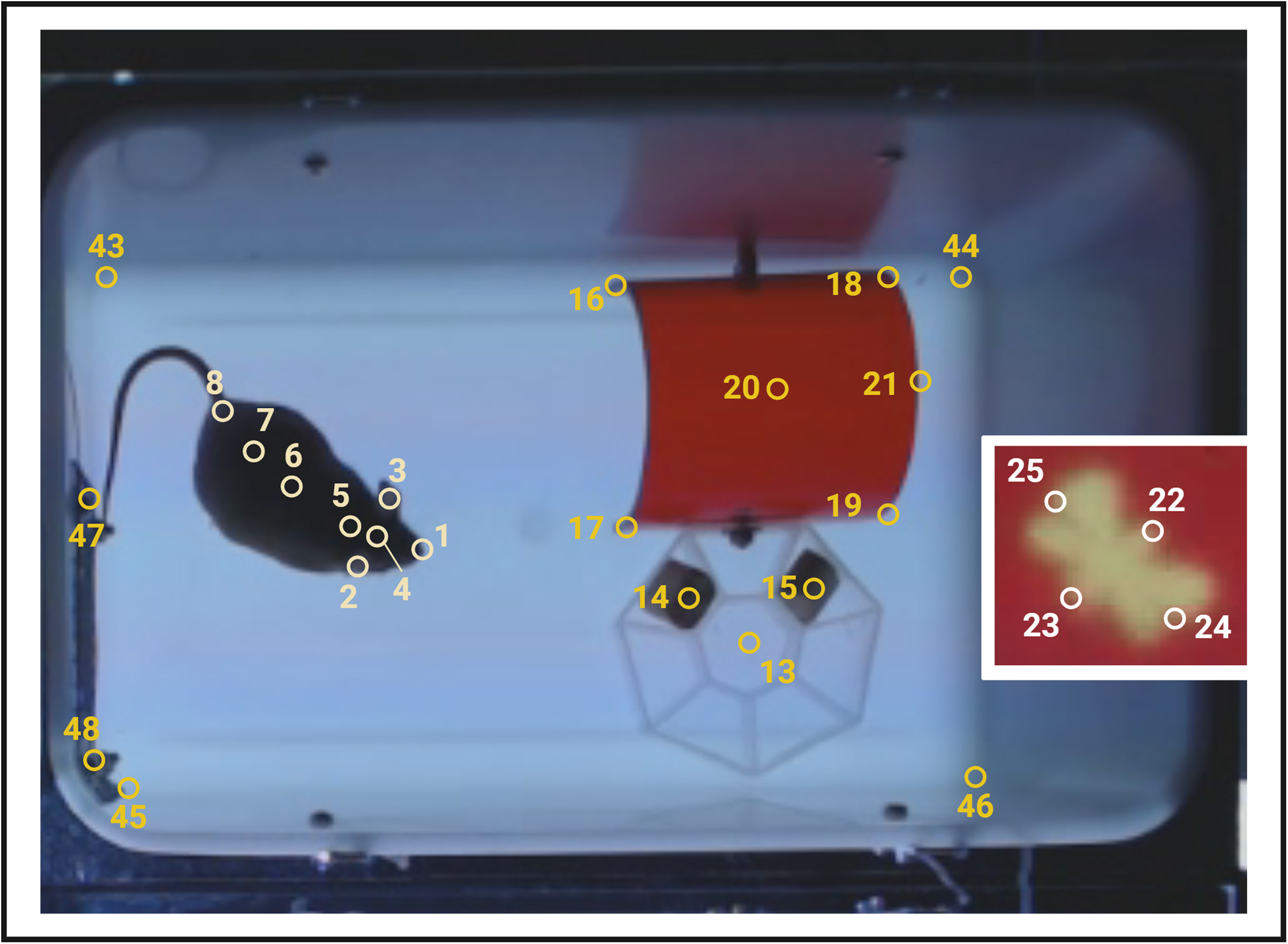

Supplement: 10 [file NIHMS2042844-supplement-10.jpg]

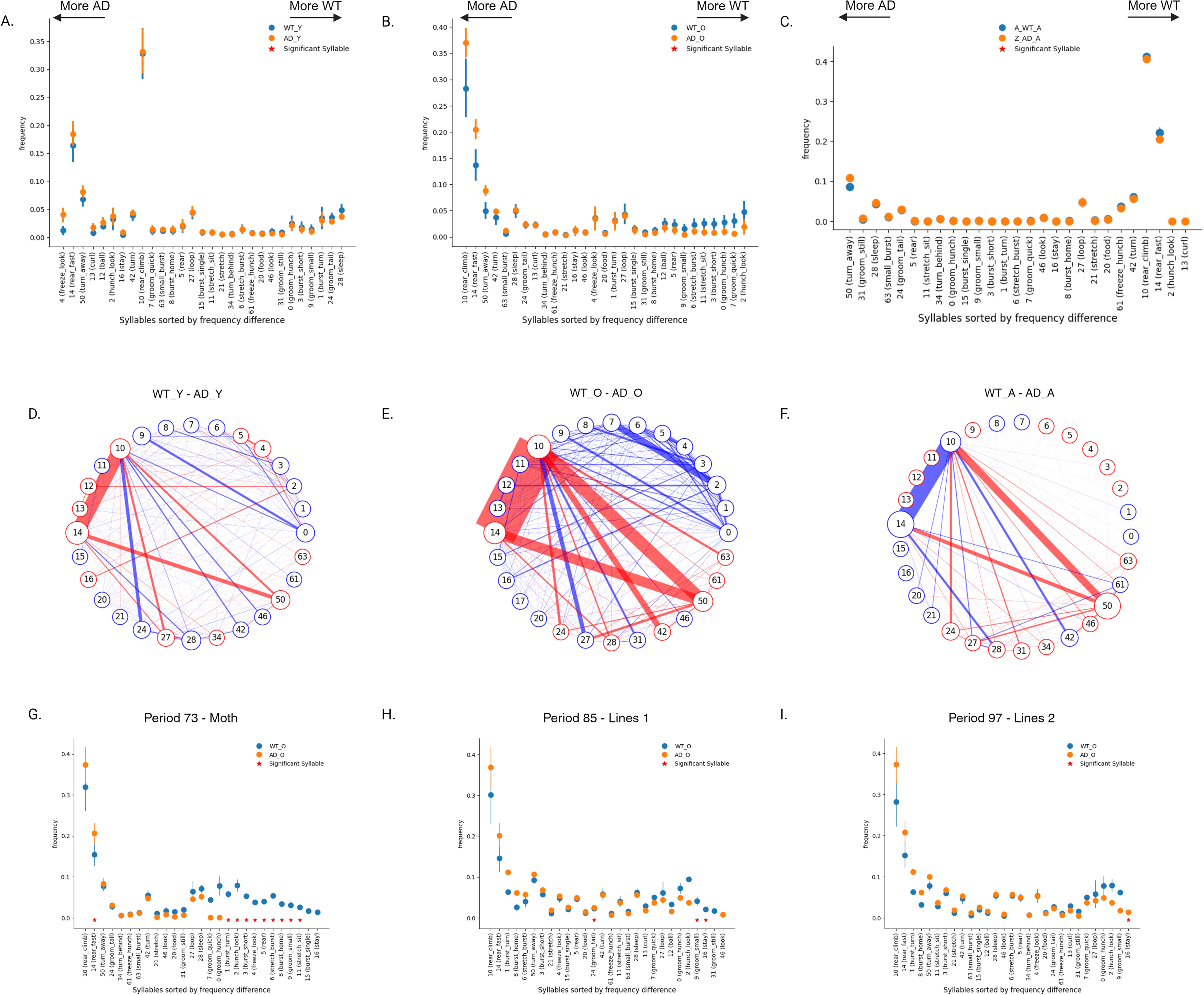

Supplement: 9 [file NIHMS2042844-supplement-9.jpg]
